# Protective effects of Schisandrin B against D-GalN-induced cell apoptosis in human hepatocyte (L02) cells via modulating Bcl-2 and Bax

**DOI:** 10.1080/21655979.2021.1979863

**Published:** 2021-09-21

**Authors:** Yanwu Hu, Haitao Li, Ruili Li, Yue Tian, Zijing Wu

**Affiliations:** a School of Medicine, Tonghua Normal University, Tonghua, China;; b Department of Pharmacy, Affiliated Hospital, Changchun University of Chinese Medicine, Changchun, China

**Keywords:** Bcl-2, Bax, schisandrin B, human hepatocyte cells, apoptosis, D-GalN-induced

## Abstract

Schisandrin B is a dibenzocyclooctadiene derivative extracted from*Schisandra chinensis* (Turcz.) Baill., that exhibits anti-oxidation, anti-inflammation, anti-tumor and hepatoprotective activities. To understand the hepatoprotective mechanism of schisandrin B, this study investigated the efficacy of schisandrin B on L02 cells after treatment with D-GalN. Following pretreatment with 40 μM schisandrin B, L02 cells were stimulated with 40 mM D-GalN. Cell viability, apoptosis, the expression levels of genes associated with apoptosis, and the intracellular oxidative stress indexes were measured. The viability of L02 cells was determined using MTT assay, and the Annexin V-FITC/PI assay kit was utilized for the assessment of apoptosis. The activities of GSH-Px and SOD, the level of MDA were assessed, separately, using relative detection kits. Moreover, RT-PCR as well as Western blot was applied to measure the mRNA and protein expression of Bax and Bcl-2. The results indicated that schisandrin B significantly prevented D-GalN‑induced oxidative damage in L02 cells (P<0.05), decreased GSH-Px and SOD activities (P<0.05), increased MDA content (P<0.05). Furthermore, schisandrin B inhibited D-GalN-induced apoptosis in L02 cells (P<0.05), regulated the expression of Bax and Bcl-2 (P<0.05). The results indicated that schisandrin B decreased the D-GalN-induced intracellular oxidative stress indexes generation, and inhibited the down-regulation of Bcl-2 and up-regulation of Bax induced by D-GalN. In conclusion, schisandrin B was shown to exert protective effect against oxidative damage of L02 cells, which, in part, was achieved by regulating the mRNA and protein levels of Bax and Bcl-2.

## Graphical Abstract

**Figure:**
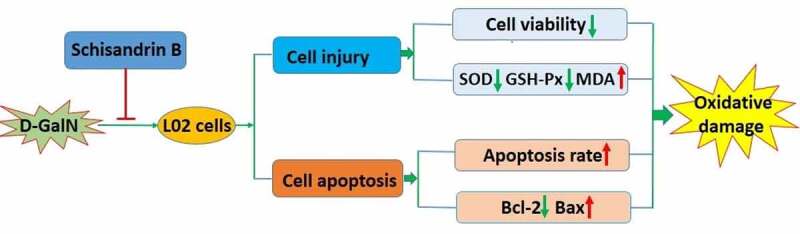

## Introduction

Apoptosis produces significant effects in many diseased and physiological conditions [1] and apoptosis of hepatocyte is known to play a key role in the emergence and the progression of a variety of acute and chronic liver diseases [2]. Excessive apoptosis has been proven to be participating in the emergence and the progression of many liver diseases, for instance, nonalcoholic liver disease, viral hepatitis as well as the alcoholic liver disease [3]. Oxidative stress has been confirmed to produce significant function in drug-induced hepatocyte apoptosis and liver injury [4]. A variety of pathogenic factors (such as hepatitis virus, alcohol, drugs, and hepatotoxicants) can break the balance of oxidative stress and eventually lead to hepatocyte apoptosis and liver damage. Studies have shown that antioxidants can reverse the pathological oxidation-antioxidant imbalance at sites of oxidative damage, thereby significantly enhancing immune cell function, and preventing oxidant-mediated tissue damage [2,5]. Hence, the issue of how to protect liver cells from apoptosis induced by oxidative stress has become pivotal to the protection of liver tissue and function.

**Figure 1.**
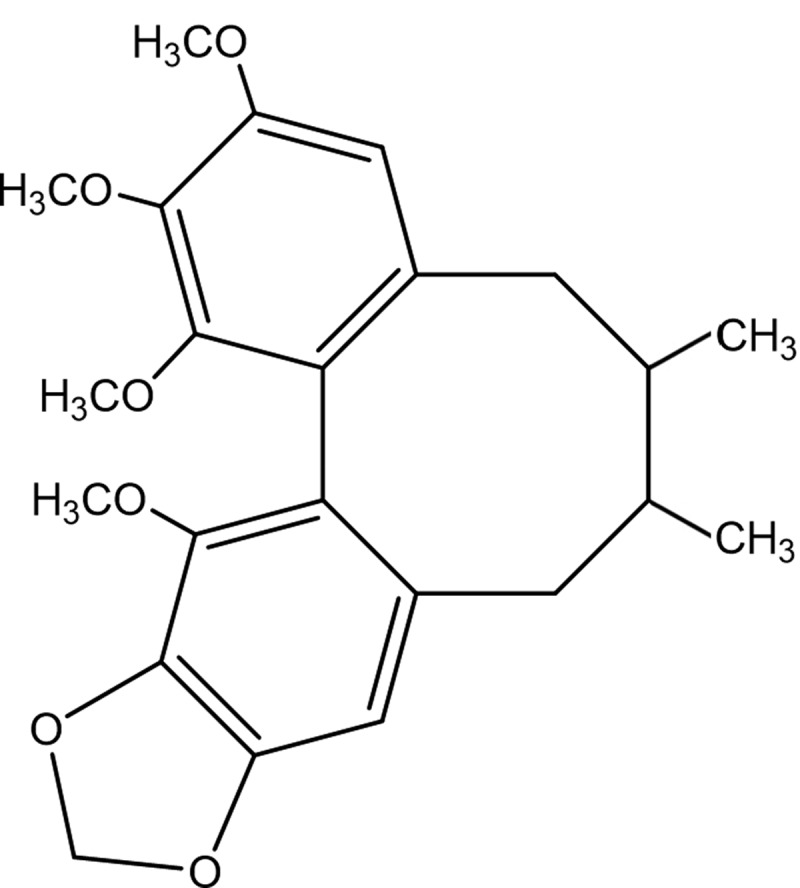
Chemical structure of schisandrin B

Schisandrin B (C_23_H_28_O_6_; Figure 1) is a pharmacologically active dibenzocyclooctadiene derivative, which extracted from the *Schisandra chinensis* fruit (Turcz.) Baill [6,7]. It is an established famous traditional Chinese medicine, which is utilized clinically as anti-hepatotoxic agent [8]. Experimental investigations have demonstrated that schisandrin B has multiple pharmacological properties including anti-oxidation, anti-inflammation [9] and anti-tumor [10]. Schisandrin B is also used for treating viral and chemical hepatitis, cellular apoptosis, liver injury mediated by immune, and hepatic fibrosis by protecting the liver from damage associated with the oxidative stress [8,11]. However, the exact mechanisms involved are yet to be elucidated.

Based on previous findings, we hypothesized that schisandrin B has a protective effect on D-galactosamine (D-GalN)-induced normal human hepatocytes (L02 cells). To test this, we investigated the efficacy of schisandrin B on D-GalN-induced injury in L02 cells by evaluating cell viability, apoptosis and its related genes or proteins including Bax and Bcl-2. This study was designed to explore how schisandrin B regulates the expression of Bcl-2 and Bax in D-GalN-induced L02 cells, with the initial aim of investigating the function of schisandrin B to protect L02 cells against D-GalN-induced injury, thereby providing a theoretical basis for the potential application of schisandrin B as a new natural hepatoprotective drug.

## Material and methods

### Cell culture and treatments

Normal human hepatocytes (L02 cells) acquired from the Cell Bank of the Chinese Academy of Sciences (Shanghai, China), and were cultured in the Dulbecco’s modified Eagle’s medium (DMEM, Gibco, USA) including penicillin/streptomycin (1%, P/S; Sigma–Aldrich, St. Louis, MO, USA), and fetal bovine serum (10%, FBS; Sijiqing Bioengineering Material Co., Ltd. Hangzhou, China) in the humidified air containing 5% CO_2_ at 37°C. After reaching 80–90% confluence, the cells were digested using 0.02% of ethylenediamine tetraacetic acid (EDTA) or 0.25% of trypsin diluted in a ratio of 1:3. 100 μM of schisandrin B (purity greater than 98%; Vic’s biological technology Co., Ltd, Chengdu, China) stock solution was generated in dimethyl sulfoxide (DMSO), and before use in the experiments, it was diluted in culture media. The final vehicle DMSO concentration was less than 0.1% in all samples.

### Cell viability assay

To determine the noncytotoxicity of schisandrin B and cytotoxicity of D-GalN on the L02 cells, the 3-(4, 5-dmethylthiazol-2-yl)-2, 5-diphenyl tetrazolium bromide (MTT; Sigma‑Aldrich) method was utilized to detect the effects of schisandrin B and D-GalN on L02 cell viability. In short, at a cell density of 1 × 10^4^ cells/well, cells could be cultured in 96-well plates and then incubated for 12 hours. After treatment with D-GalN and/or schisandrin B for a specified time, the cells were incubated in 0.5 mg/mL of MTT reagent for four hours, and discarding supernatant, and then harvested by dissolving the precipitate, in dimethyl sulfoxide (150 μL). At 570 nm wavelength, the microplate reader what could be used to monitor absorbance (Thermo Scientific, Rockford, IL, USA). Cell viability was calculated as the percentage of cell absorbance between the sample and the control (medium only) [12].

### Flow cytometry analysis of apoptosis

The level of apoptosis was assessed using an Annexin V-FITC/PI kit (Shanghai Sangon Biotech Co., Ltd., China) and FACSCalibur (Becton Dickinson, San Jose, CA, USA). L02 cells were cultured into 6-well plates at a cell density of 1 × 10^4^ cells/well and then incubated for 12 h. After being exposed to the required experimental conditions, cells were collected and then cleaned using a binding buffer. Afterward, the cells were suspended in the binding buffer (195 μL) at a cell density of 2 × 10^5^ cells per mL, and then incubated in Annexin V-FITC (5 μL) in dark at ambient temperature for 15 min. After incubation, the binding buffer (200 μL) was used to wash L02 cells, and the cells were centrifuged for 5 min at 1000rpm. The cells were suspended in the binding buffer (190 μL) containing 10 μL PI. The software of FlowJo 7.6 software was utilized to analyze the percentages of apoptotic cells [13].

### Measurement of intracellular oxidative stress indexes

At a cell density of 1 × 10^4^ cells/well, L02 cells were cultured in 6-well plates and incubated for 12 hours. After exposure to the required experimental conditions, the supernatant was carefully removed, and the cells were harvested. Then, cells were lysed in an ultrasonic processor and centrifuged for 10 minutes at 10,000 rpm, followed by the determination of the total protein concentration, the content of intracellular MDA, the GSH-Px and SOD activities of the supernatant. The procedures for determination were based on manufacturer protocols for the MDA, GSH-Px and SOD detection kits (Nanjing Jiancheng Bioengineering Institute, China) [14].

### Western blot analysis

At a cell density of 1 × 10^4^ cells/well, L02 cells were cultured in 6-well plates and they were incubated for 12 h. After exposure to the required experimental conditions, the cells were harvested and then lysed on ice by using RIPA lysis buffer (Beyotime, Jiangsu, China). The cell extracts protein concentration was detected via applying the bicinchoninic acid (BCA) protein assay kit (Beyotime Biotech, Shanghai, China). Equal protein samples were placed in 10% gels of sodium dodecyl sulfate-polyacrylamide gel electrophoresis (SDS-PAGE) (1.5 hours, 120 V), then they were transferred to membranes of polyvinylidene difluoride (PVDF) (one hour, 100 V), and blocked in the Tris-buffered saline containing 0.1% Tween-20 (TBST) buffer at ambient temperature by using 5% skimmed milk for one hour. After blocking, the specific primary antibodies and membranes were cultured overnight at 4°C: anti-Bax (1:1,000; cat. no. sc-8102; Santa Cruz Biotechnology, Santa Cruz, CA, USA), anti-Bcl-2 (1:1,000; cat. no. sc-8024; Santa Cruz Biotechnology, Santa Cruz, CA, USA), anti‑GAPDH (1:1,000; cat. no. sc-6341; Santa Cruz Biotechnology, Santa Cruz, CA, USA), then washed prior to incubation with an appropriate horseradish (1:5,000; cat. no. sc-5203; Santa Cruz Biotechnology, Santa Cruz, CA, USA) were visualized through strengthened the chemiluminescence (ECL, Amersham Biosciences Corp, NJ, USA), and the analysis of relative band intensity was conducted with ImageJ version 1.46 (National Institutes of Health, Bethesda, MD, USA) [15].

### Real time-PCR analysis

At a cell density of 1 × 10^4^ cells/well, L02 cells were cultured in 6-well plates and then incubated for 12 hours. After exposing cells to the required experimental conditions, the overall RNA was extracted by using the reagent of Trizol (Takara Biomedical Technology Co., Ltd., China) according to manufacturer instructions of cDNA was synthesized via reverse transcription utilizing the PrimeScript RT Master Mix kit (Takara Biotechnology Co., Ltd., Dalian, China) based on the manufacturer protocol, with the conditions of 85°C for 5 min and 37°C for 15 min, after which, quantitative real time-PCR was performed using the gene-specific primers of SYBR® Premix Ex Taq (Beijing Transgen Biotech Co., Ltd., Beijing, China) with these conditions: pre-denaturization for 5 min at 95°C, then 40 cycles of denaturization for 10 s at 95°C, followed by annealing for 15 s at 60°C, and extending for 30 s at 72°C, ultimately extending for 10 minutes at 72°C. The following oligonucleotide primers were used: Bax forward: 5ʹ-CCCGAGAGGTCTTTTTCCGAG-3ʹ, reverse: 5ʹ-CCAGCCCATGATGGTTCTGAT-3ʹ; Bcl-2 forward: 5ʹ-GGTGCCACCTGTGGTCCACCT-3ʹ, reverse: 5ʹ-CTTCACTTGTGGCCCAGATAGG-3ʹ; and GAPDH forward: 5ʹ-GTTACCAGGGCTGCCTTCTC-3ʹ, reverse: 5ʹ-GATGGTGATGGGTTTCCCGT-3ʹ. For each sample, mRNA level was normalized to the GAPDH level; all experiments were performed in triplicate. The relative quantification analysis used was the 2^−ΔΔCt^ method [16].

### Statistical analysis

The statistics were implemented with the program of SPSS version 19.0 (Chicago, IL, USA). Each independent experiment had been repeated at least three times, and the analysis of data could be performed via the one-way analysis of variance (ANOVA) and then the Tukey’s post hoc test was used to conduct multiple comparisons [17]. Results are represented as mean ± SEM, and the criterion for statistical was *P* < 0.05.

## Results

To investigate the hepatoprotective mechanism of schisandrin B, we used L02 cells stimulated with D-GalN to establish an *in vitro* cell model. We investigated the effects of schisandrin B on cell viability, apoptosis, GSH-Px and SOD activities, MDA content, as well as the mRNA and protein expression of Bax and Bcl-2. We found that schisandrin B had a robust beneficial protective effect against D-GalN-induced injury in L02 cells. Notably, we demonstrated that these effects are mediated by oxidative stress response and apoptosis.

### Schisandrin B protects L02 cells against D-GalN induced injury

**Figure 2.**
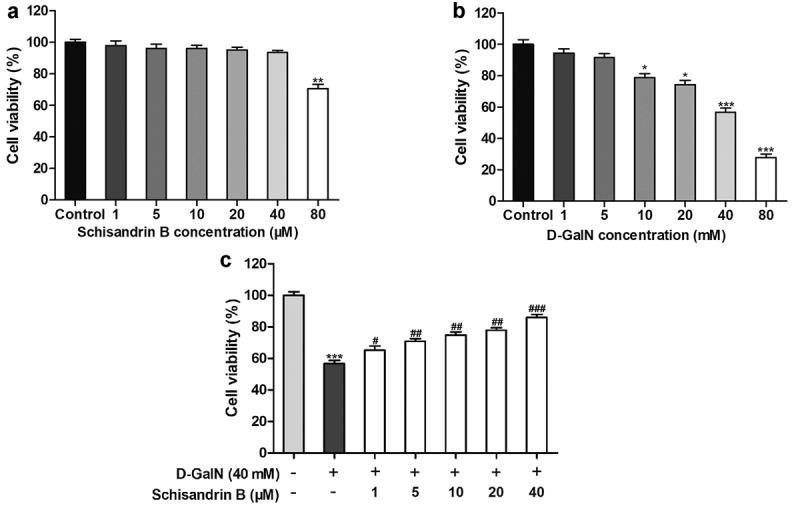
Protective effects of schisandrin B on D-galactosamine (D-GalN)-induced cytotoxicity in L02 cells. (a) Cells were treated with D-GalN (0, 1, 5, 10, 20, 40, 80 mM) (b) or schisandrin B (0, 5, 10, 20, 40, 80 μM) for 12 h. (c) Cells were pre-treated with schisandrin B (0, 1, 5, 10, 20, 40 μM) for 12 h followed by D-GalN (40 mM) treatment for 12 h. Cell viability was detected by using the MTT reagent. Data are expressed as mean±SEM. Compared with control, *P < 0.05, **P < 0.01, ***P < 0.001; compared with D-GalN, ^#^*P* < 0.05, ^##^*P* < 0.01, ^###^*P* < 0.001

To study the protective effect of schisandrin B on the D-GalN-induced injury in L02 cells, we first measured the concentrations responses range of schisandrin B effects that were not. L02 cells cultures were treated with schisandrin B concentrations ranging from 1 to 80 μM for 12 hours, and then accessed cell viability the MTT assay. We showed that cells remained viable when treated with schisandrin B concentrations of 1–40 μM, but that cell viability was reduced to 70.71% ± 6.04% when treated with 80 μM schisandrin B (Figure 2(a)). Hence, in subsequent experiments, the maximal schisandrin B concentration used was 40 μM. Next, to assess the cytotoxicity of D-GalN, after incubation in D-GalN concentrations ranging between 1 and 80 mM for 12 hours, cell viability decreased in a concentration-dependent manner. After treated by the D-GalN concentration of 40 mM for 12 hours, decreased cell viability to 56.78% ± 5.76% (Figure 2(b)). As a result, this concentration was used in all following experiments assessing the potential cytoprotective effect of schisandrin B. Next, to study the schisandrin B’s protective effect, L02 cells were first pretreated with schisandrin B (1 to 40 μM) for 12 hours, and then treated with 40 mM D-GalN for 12 hours. The results obtained (Figure 2(c)) indicated that in the D-GalN group, cell viability was remarkably lower than that of the control group, and that at concentrations from 1 to 40 μM, schisandrin B prevented the injury induced by the D-GalN in a concentration dependent manner. Hence, this suggests that pretreatment by schisandrin B (40 μM) for 12 hours and then incubating in 40 mM D-GalN for 12 hours produced the best condition for the following experiments.

### Protective effects of schisandrin B against D-GalN induced apoptosis

**Figure 3.**
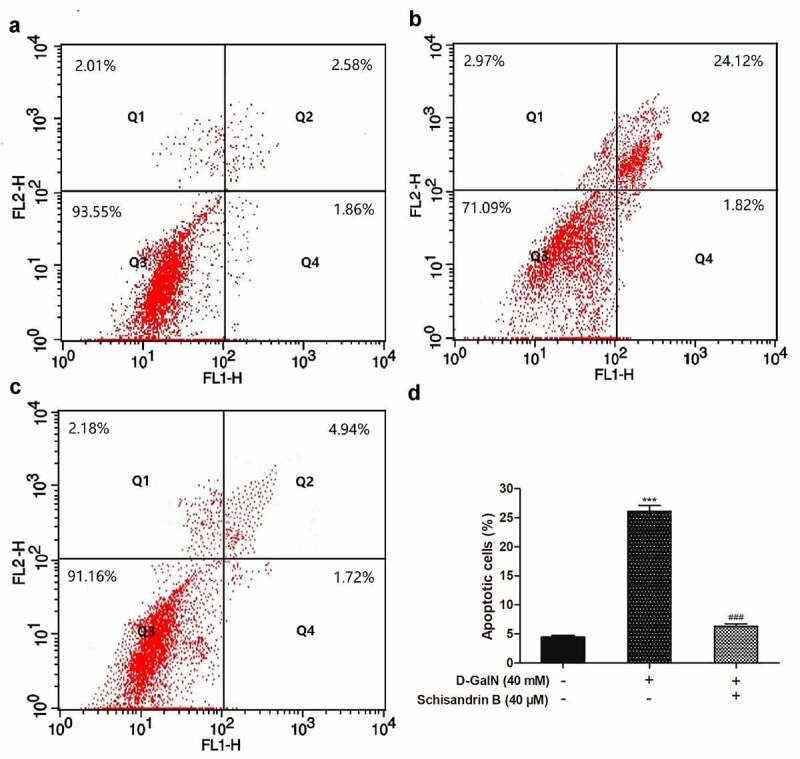
Anti-apoptotic effects of schisandrin B on D-GalN-induced L02 cells. (a) Control group; (b) Cells treated with D-GalN (40 mM) alone; (c) Cells pre-treated with schisandrin B (40 μM) for 12 h and then treated with D-GalN (40 mM) for 12 h; (d) The results of the apoptotic rate. The rate of apoptosis was evaluated by flow cytometry. Data are expressed as mean±SEM. Compared with control, ***P < 0.001; compared with D-GalN, ^###^*P* < 0.001

To determine the schisandrin B effect on L02 cell apoptosis, the apoptosis rate was investigated using flow cytometry and the Annexin V-FITC/PI assay. As illustrated in the (Figure 3), the early apoptotic rate is in the Q4 region, while the late apoptosis rate is in the Q2 area. D-GalN significantly increased the apoptotic rate of L02 cells. Nevertheless, in comparison with the D-GalN group, schisandrin B evidently decreased the increase of apoptosis rate. These findings demonstrated that schisandrin B strongly inhibited apoptosis in the L02 cells stimulated by D-GalN.

### Protective effects of schisandrin B against D-GalN Induced Cellular Oxidative Damage

**Figure 4.**
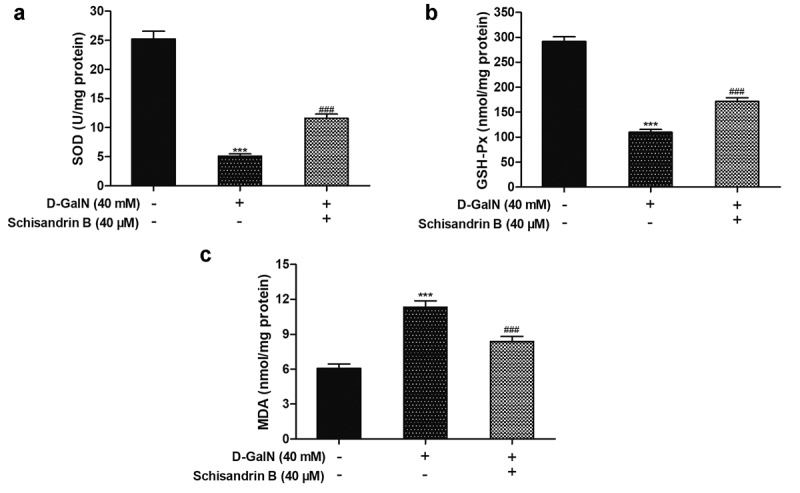
Effects of schisandrin B on oxidative stress indexes in D-GalN-induced L02 cells. (a) The relative SOD activity; (b) The relative GSH-Px activity; (c) The relative MDA content. Data are expressed as mean±SEM. Compared with control, ***P < 0.001; compared with D-GalN, ^###^*P* < 0.001

To study if schisandrin B mediates antioxidant enzyme activities, the amout of MDA, GSH-Px and SOD activities in D-GalN-induced L02 cells were measured. Compared with the control group, shows that GSH-Px and SOD activities were reduced in the D-GalN-treated L02 cells, and that the content of MDA was increased, whereas treatment with schisandrin B significantly inhibited these effects (Figure 4). These outcomes showed that schisandrin B inhibits the oxidative damage in L02 cells induced by D-GalN.

### Regulatory effects of schisandrin B on protein and mRNA expression levels of Bax and Bcl-2 in D-GalN-induced L02 cells

**Figure 5.**
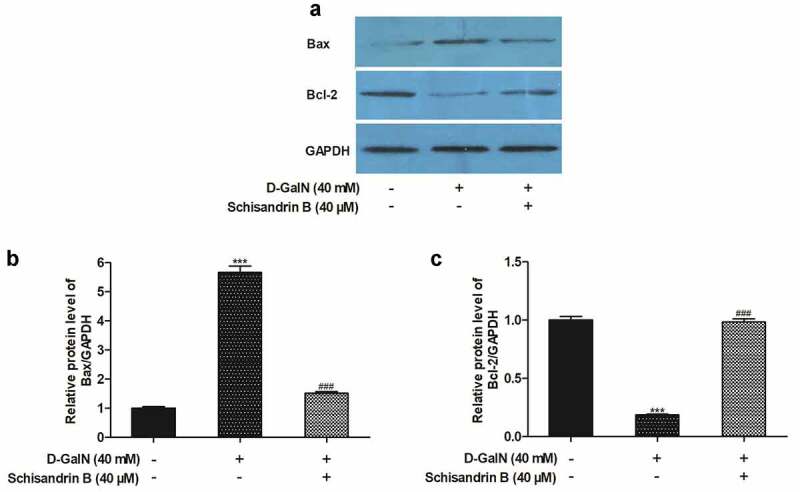
Effects of schisandrin B on the protein expression of Bax and Bcl-2 in D-GalN-induced L02 cells. (a) Representative immunoblots for the Bax and Bcl-2, and GAPDH proteins; (b, c) The relative protein expression levels of Bax/GAPDH, Bcl-2/GAPDH. The data on quantified protein expressions were normalized by related GAPDH (fold change of control). The protein expression was detected by Western blot analysis. Data are expressed as mean±SEM. Compared with control, ***P < 0.001; compared with D-GalN, ^###^*P* < 0.001

**Figure 6.**
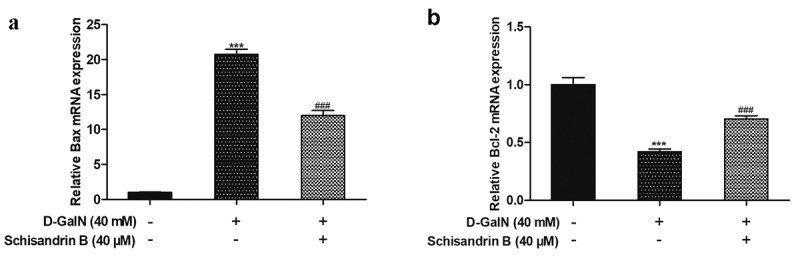
Effects of schisandrin B on the mRNA expression of Bax and Bcl-2 in D-GalN-induced L02 cells. (a) Bax mRNA expression and the ratio to the control level; (b) Bcl-2 mRNA expression and the ratio to the control level; (c) The mRNA expression was detected by RT-PCR; as a loading control, GAPDH was used. Data are expressed as mean±SEM. Compared with control, ***P < 0.001; compared with D-GalN, ^###^*P* < 0.001

To investigate the schisandrin B’s protective mechanism on apoptosis in L02 cells induced by D-GalN, we conducted detailed studies of the expression levels of Bax and Bcl-2 related proteins and genes by Western blot analysis and real-time reverse transcription-polymerase chain reaction. The results revealed that D-GalN enhanced the mRNA and protein expression levels of Bax but reduced the Bcl-2 expression in the L02 cells (Figures 5**–**6). In comparison with D-GalN group, in schisandrin B group, the expression level of Bax was reduced, and the Bcl-2 expression level was increased. These results suggested that schisandrin B can reduce apoptosis in D-GalN-induced L02 cells with the down-regulation of Bax mRNA and protein expression levels and the up-regulation of Bcl-2 mRNA and protein expression levels.

## Discussion

D-GalN is an established hepatotoxicity experimental drug, and D-GalN-induced hepatocyte injury models have been employed, extensively, in research investigating the pathogenesis of liver injury and hepatoprotective drugs [4]. Studies have confirmed that the progression of liver injury is related to oxidative stress, while D-GalN leads to cellular oxidative damage [18]. D-GalN can destroy intracellular the oxidative stress balance and defense systems of primary antioxidants containing the redox cycle of GSH and SOD [19,20]. SOD is an important antioxidant enzyme, that can catalyze the conversion of superoxide radical into H_2_O_2_, then continuously detoxify them to H_2_O [21–23]. Therefore, SOD protects hepatocytes from the harmful effects caused by superoxide radicals to a certain degree. The redox cycle of GSH regulates redox reactions in hepatocytes induced by intracellular and extracellular stimulation. GSH is one of the major components of GSH redox cycle. It is a cofactor of scavenging alkyl peroxides, lipid peroxides, and H_2_O_2_. It is responsible for scavenging hydroxyl radicals and regulating the intracellular redox balance. GPx, another major component of the GSH redox cycle, reduces lipid hydroperoxides to form corresponding hydrogen peroxide and alcohols in the liver. As noted above, oxidative stress exerts a significant effect on hepatocyte injury induced by D-GalN, the progression of liver injury was associated with the level of oxidative stress. As a result, MDA, the lipid peroxidation toxic product, is also an important biomarker of oxidative injury [24,25]. Hepatotoxic drugs or harmful substances usually increase the concentration of MDA in liver tissue, thereby influencing the activities of antioxidant enzymes [26]. In this study, our results demonstrate, for the first time, that schisandrin B protected L02 cells from the cell injury induced by D-GalN. The schisandrin B’s effects related to increased apoptosis. Treatment by D-GalN significantly decreased the viability of L02 cells, and increased the apoptotic rate, while schisandrin B significantly reversed the D-GalN mediated effects in L02 cells. In addition, our study indicates that the D-GalN cytotoxicity is mediated by intracellular oxidative stress and demonstrated schisandrin B’s protective effect as an antioxidant against cytotoxicity induced by D-GalN in the L02 cells.

While aiming to elucidate the mechanism underlying the protective effects of schisandrin B on apoptosis via the D-GalN in L02 cells, we also assessed Bax and Bcl-2 at both the protein level and gene level. Previous studies have shown that Bcl-2 family members have a significant effect on the regulation of apoptosis [2,27]. Members of Bcl-2 family are either antiapoptotic or proapoptotic. Among them, the anti-apoptotic protein Bcl-2 and pro-apoptotic protein Bax are viewed as being essential for determining whether the process of apoptosis occurs [28]. It has been identified that oxidative stress challenge downregulated of Bcl-2 and upregulated of Bax in L02 cells [2]. In this current study, it can be found that D-GalN remarkably upregulated Bax and downregulated Bcl-2 at the protein and mRNA levels. Schisandrin B also played a preventive role in protein and mRNA levels, and schisandrin B inhibited the up-regulation of Bax and down-regulation of Bcl-2 induced via the D-GalN. These results exhibited that the Bcl-2 family proteins may have an essential effect in the regulation of the apoptosis induced via the D-GalN in the L02 cells, while schisandrin B inhibits the apoptosis induced via the D-GalN with the regulation of Bax and Bcl-2 at the protein and RNA levels.

In summary, our study demonstrated that schisandrin B’s protective effects on the L02 cells from D-GalN-mediated apoptosis induced via D-GalN. These protective effects are related to D-GalN-mediated oxidative stress, up-regulation of Bax and down-regulation of Bcl-2. These results provide new experimental evidence that schisandrin B can prevent the development of hepatocyte injury, and supporting the potential of schisandrin B as a hepatoprotective drug targeting liver failure.

## Conclusion

In conclusion, this study revealed that schisandrin B can attenuate D-GalN-induced apoptosis and oxidative damage in L02 cells via a mechanism potentially related to the regulation of Bax and Bcl-2.

## Abbreviations

L02 cells normal human hepatocytes; D-GalN D-galactosamine; EDTA ethylenediamine tetraacetic acid; FBS fetal bovine serum; DMEM dulbecco’s modified eagle medium; P/S penicillin/streptomycin; DMSO dimethyl sulfoxide; MTT 3-(4, 5-dmethylthiazol-2-yl)-2, 5-diphenyl tetrazolium bromide; MDA malondialdehyde; GSH-Px glutathione peroxidase; SOD superoxide dismutase; BCA bicinchoninic acid; SDS-PAGE sodium dodecyl sulfate-polyacrylamide gel electrophoresis; PVDF polyvinylidene fluoride; TBST tris-buffered saline containing 0.1% Tween-20; GAPDH glyceraldehyde-3-phosphate dehydrogenase; PCR polymerase chain reaction; mRNA messenger RNA; ANOVA one-way analysis of variance; Bcl-2 an anti-apoptotic protein; Bax an apoptotic protein.

## Highlights

1. Schisandrin B inhibited D-GalN-induced apoptosis in L02 cells.
2. Schisandrin B prevent D-GalN-induced oxidative in L02 cells.
3. The regulatory effects of schisandrin B on Bax and Bcl-2 in D-GalN-induced L02 cells.
